# Changes in school-day step counts during a physical activity for Lent intervention: a cluster randomized crossover trial of the Savior’s Sandals

**DOI:** 10.1186/s12889-019-6479-9

**Published:** 2019-02-01

**Authors:** David Kahan, Kent A. Lorenz, Eyad Kawwa, Andrew Rioveros

**Affiliations:** 1 School of Exercise and Nutritional Sciences, San Diego State University, 5500 Campanile Drive, San Diego, CA 92182 USA; 2 Department of Kinesiology, San Francisco State University, 1600 Holloway Avenue, San Francisco, CA 94132 USA

**Keywords:** Catholicism, Lent, Physical activity, Middle school, Pedometer

## Abstract

**Background:**

Participation in regular physical activity (PA) has many health benefits for school-aged children. However, only about 20% of children worldwide meet recommendations for being sufficiently active. Children spend many hours per day at school and schools have a mandate for promoting PA. Private religious schools could serve as a novel source for religious-themed PA interventions.

**Methods:**

We randomly allocated 2 clusters of 2 Catholic middle (grades 6–8) schools/cluster to a 2-week no treatment/4-week intervention crossover trial to determine the effects of a 20-day Lenten-themed PA intervention on 187 students’ pedometer steps taken at school. Specifically, students independently progressed through a workbook (*Savior’s Sandals*) that depicted and informed about 11 locations in the Holy Land where Jesus lived, visited, and/or ministered, and included Scripturally-based questions about each place for students to answer. In all, students would accumulate 110,000 steps if they completed the workbook virtual journey. General linear mixed models with restricted maximum likelihood estimation to compensate for missing data were used to compute the intervention effects on mean daily steps.

**Results:**

There were significant main effects for the intervention overall and by school and grade level. Follow-up tests isolated that a single school (Mean_diff_ = + 2156 steps/day) and grade 6 students (Mean_diff_ = + 1678 steps/day) across all four schools experienced the greatest treatment effects.

**Conclusions:**

Religious-themed PA interventions can be effective; however, specific adjustments may be needed to optimize the intervention’s effectiveness for a broader population of students.

**Trial registration:**

Current Controlled Trials ISRCTN10273669. Retrospectively registered 23 Oct 2018.

## Background

Globally, only 19% of school-going children (between ages 10–17 years) in 2010 were sufficiently physically active (i.e. accumulated ≥60 min/day of moderate-to-vigorous intensity physical activity (MVPA) or its equivalent; [1]). Insufficient levels of physical activity (PA) increase the risk of coronary heart disease, type 2 diabetes, and breast and colon cancers, and premature death in adulthood [2]. In contrast, sufficient levels of PA during youth enhance motor development, health- and performance related fitness, psychosocial and mental health, and reduce the risk of metabolic syndrome [3].

Based on accelerometry measures of PA, only 7.5% of U.S. children between ages 12–15 years (spanning the ages of students in the present study) were classified sufficiently active (i.e. ≥300 min/week of moderate intensity PA or its equivalent; [4]). Even light PA (i.e. < 3.0 MET), when accumulation of appropriate levels of MVPA is not possible, is preferable to sedentary activity among youth and may confer different effects on health than MVPA [3]. For stemming the rise of childhood physical inactivity, schools are recognized worldwide as occupying a key role in promoting PA [5]. In the United States (US), physical education (PE), particularly, is considered the foundational element of a comprehensive school PA program (CSPAP) for providing students the majority of 60 min/day of PA [6].

California private schools (location of and school type in the present study) that offer middle school grades must offer PE in accordance with California Educational Codes [7]. The law, however, does not guarantee that PE is taught well or at all, and whether it is of high quality and provided at an appropriate volume (min/week). Thus, for students to achieve recommended levels of PA at school, a whole-of-school (WOS) approach to PA curriculum was developed, which promotes PA opportunities outside PE class—including recess, classroom PA time, active transport, before- and after-school programming, and intramural and extramural sports [3]. Researchers using a WOS index (scored 0–6), comprised of six school practices for promoting/providing PA, found that 66% of middle schools in a national sample scored ≤4 [8]. Thus, such schools have room for improvement and should identify missing practices to increase students’ PA level.

The 2016 National Physical Activity Plan (NPAP) identifies 9 sectors for targeting strategies and tactics to increase PA including schools and faith-based organizations [9]. In a comprehensive review of 27 faith-based PA interventions, Bopp et al. [10] argued for the potential of religious programs and spaces to positively contribute to PA behavior. They noted, however, that only one reviewed study targeted youth and subsequently recommended that future studies include them. Among church-attending youths in the same age range, religion, and ethnicity considered in the present study (i.e 11–13 years, Catholic, Hispanic), majorities perceived some connection between faith and health and the need for churches to provide PA interventions [11]. Thus, it is reasonable to infer that youth who attend Catholic schools, should hold similar feelings.

Catholic schools are a natural nexus of the two aforementioned NPAP sectors in that there is often a physical intermingling between school personnel and students, and clergy, laity, and parishioners. Thus, PA curriculum imbued with religious elements may be well-received by all. In 2015–2016, 7000 Catholic schools and their 1.9 million students represented 20 and 39% of all private U.S. schools and private school students, respectively, and 67 and 78% of all US schools with a religious orientation and corresponding students [12]. Further, for middle school grades 6–8, Catholic schools enrolled 436,000 students in academic year 2015–2016 [13]. Therefore successful PA interventions conducted in Catholic schools that are scalable and disseminated could greatly impact public health.

We chose the Lenten period (40 days [6 school weeks] leading up to Easter) as an appropriate time during the school year to test a faith-based intervention for increasing PA at school. Kahan and Nicaise [14] conducted a conceptually similar study with Muslim middle school students and recommended synchronizing future studies with important observances on the religious calendar. They found that 7th graders – but not 6th and 8th graders – significantly increased steps during a 4-week treatment period compared to 1-week baseline (+ 850 steps) and post intervention (+ 1300 steps) conditions, while boys responded more strongly to treatment than girls (+ 600 vs. -200 steps).

We adapted the program described by Kahan and Nicase [14] to a Christian context in conjunction with the places Jesus visited (i.e. walking/steps = PA) during his lifetime leading to his death and resurrection on Easter (i.e. intervention specifically linked to a seminal religious figure and event). Our study’s main purpose was to determine whether a virtual pilgrimage that Catholic school students track by workbook and pedometers during Lent would result in increased PA vis-à-vis accumulated steps at school. Secondary purposes included determining if intervention effects differed based on gender, grade level, and treatment order.

## Methods

### Schools

**Fig. 1:**
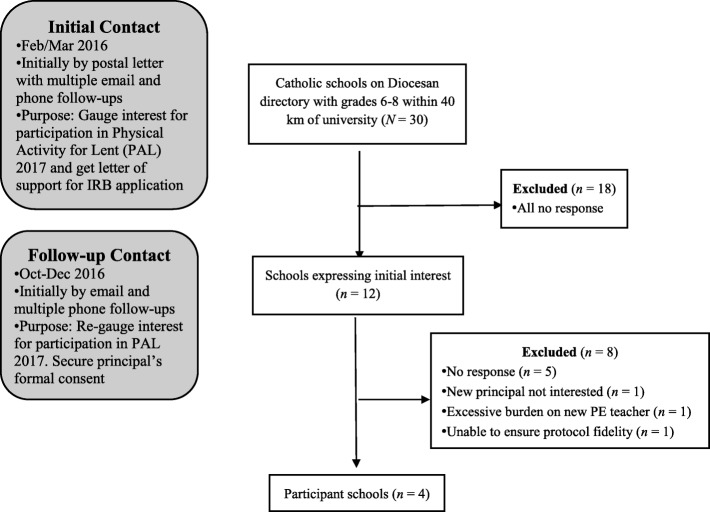
Flow chart of sampling frame to study sample

We developed a sampling frame of 30 Catholic schools (see Fig. 1) with middle school grades 6–8 listed on the San Diego Diocesan register of schools and located within a 40-km radius of the university. In February/March 2016 (last 3 weeks of Lent), we sent the principal of each school a letter introducing the study and indicated we would attempt to contact them in a week. We phoned and emailed principals up to three times to establish initial contact resulting in 12 principals showing initial interest in the study. Beginning in October 2016, we re-contacted these principals to check if they were still interested. Ultimately, four principals – in consultation with clergy and staff – formally consented their school’s participation.

Table 1 presents school characteristics. School 1’s 8th grade students did not participate due to low interest resulting in a sampling frame of 202 students with a 93% participation rate. (The sample was 59% female.) The percentage of the neighborhood population that is Hispanic (Table 1) reflects the census tract data associated with school location, because we did not ask participants or principals for ethnic/racial identity data. Based on surname and physiognomy, however, participants at Schools 1, 3, and 4 were primarily Hispanic and at School 2 were primarily Non-Hispanic white. (School 4 had a much higher percentage of Asian students than any other school.) A PE specialist at School 1, YMCA staff at Schools 2 and 3, and 2–3 sports coaches at School 4 taught PE lessons twice weekly.

**Table 1:** Select characteristics of participating schools (*N* = 4)

School	First allocated to	Grade 6–8 population (*N*)	Participants (*n*)	Female (%)	Neighborhood Hispanic (%)
1	Treatment	43^a^	35	51.4	52.6
2	Treatment	52	50	54.0	24.3
3	Control	43	43	83.7	63.3
4	Control	64	59	45.4	34.8
Total/Mean		202	187	58.7	49.7

*Note*. Superscript ^a^ refers to only 6th and 7th graders participating in the study because—according to school staff—there was insufficient interest among 8th graders

### Conceptual underpinnings of the intervention

Several propositions informed the *Savior’s Sandals* intervention. We envisioned the program as an exercise in mindfulness (focusing on the present and being aware of behavior as it happens) during the Lenten season of 40 days—preparatory to Easter’s celebration of Christ’s resurrection. During this time some people choose to mindfully add a healthy behavior to their lives in lieu of or in addition to abnegation. Promotion of mindfulness in health behavior interventions is recommended as it is associated with various beneficial psychological states (e.g. self-efficacy, adherence) related to exercise behavior [15]. Individuals who acted mindfully were more likely—than their less mindful counterparts—to fulfill their intention to exercise, which researchers attributed to “the ability to stay focused on the fulfillment of plans and control counter-intentional thoughts that often detract people from acting on their intentions” ([16] p. 671). In the context of PA and Lent, when presenting the program to school personnel and students, we referred to Jesus’s ambulatory behavior:

Jesus *walked*. To make the customary three trips to Jerusalem each year to keep the holy days. …Jesus would have walked about 150 miles round-trip on each occasion. … When you consider that He probably walked a mile or more a day during the rest of the year, it is not hard to see that Jesus could have easily walked more than 1000 miles every year! ([17] p. 1).

We specifically asked students to consider ‘What would Jesus do?’ when faced with a decision to be sedentary or active during the school day. If/when utilized by targeted group members this reflective question and its answer presupposed an element of faith. According to the National Study of Youth and Religion, 86% of US Catholic teenagers believed that religious faith is somewhat, very, or extremely important in daily life [18].

Based on Fowler’s Stages of Faith Theory we expected participants to occupy the Mythic-Literal or Synthetic-Conventional stage of faith development [19]. In the former (stage 2), school-aged children typically accept the stories told to them by their faith community but interpret these stories literally, whereas in the latter (stage 3), teenagers adopt some form of all-encompassing belief system by coalescing personal, friend, family, and community beliefs [19]. It is in Catholic schools’ purview to “unlock the intellectual potential of the young people they serve while forming them for a personal and living encounter with Jesus Christ” ([20] p. 3). Moreover, the Catholic Church in the US specifically enjoins its schools to teach as Jesus did, which in part focuses on inculcation of God’s revelatory message exemplified in the life of Jesus [21]. For US Catholic schools the Lenten season provides opportunities for additional programming that are not available the rest of the school year such as weekly Stations of the Cross and the Catholic Relief Fund’s national Operation Rice Bowl (https://www.crsricebowl.org/). Thus, Catholic school students may expect added programming (i.e. *Savior’s Sandals*) during Lent.

We also considered elements of social cognitive theory (SCT) [22]. Self-regulatory factors such as goal setting and self-monitoring were included in program presentations to students and incorporated into workbook and step log materials. Social support is another element of SCT with friends’ encouragement, own PA, and co-participation positively associated with children’s PA [23]. We initially stressed that participants should individualize step count goals and that the program was non-competitive. Yet we knew that pedometer-based PA programs elicit social comparison and competition [24], so we also recommended to students to seek out friends to be physically active with at school to help achieve their goals. Self-efficacy—a third element of SCT—is positively associated with objectively measured PA in this age group [25], and applied to the *Savior’s Sandals* refers to one’s confidence to increase steps during Lent (i.e. the outcome) based on perceived motor competence (task efficacy) and ability to overcome barriers (barrier efficacy). Accordingly, we drew upon the verbal persuasion source of self-efficacy to stress that everyone has the ability to step, that the average number of steps necessary to complete the virtual journey was achievable with effort, and identified several opportunities to accumulate additional steps during a typical school day.

### Design and materials

The schools’ principals agreed to participate on the condition that their students would engage with the *Savior’s Sandals* workbook. Thus, we could not employ a control group. Instead, we utilized a crossover design whereby we randomly assigned two schools to control conditions for the first 2 weeks of Lent followed by treatment conditions for the remaining 4 weeks of Lent. The remaining two schools proceeded in reverse order. Thus, over the 6-week period the schools followed either an AABBBB or a BBBBAA design.

During control conditions, we furnished students with a single page log for them to numerically record and bar graph daily step counts. During treatment conditions, we additionally provided each student with the *Savior’s Sandals* workbook. We conceptualized the workbook after one used in a study of Muslim middle school students’ step counts during a virtual pilgrimage to Mecca, Saudi Arabia [14]. An ordained Christian minister translated the aforementioned workbook into an 11-location virtual journey of Jesus Christ’s life path from Bethlehem to Jerusalem whose completion required students to take 110,000 steps in 4 weeks. Each location (e.g. Egypt, Nazareth, Cana) was printed on its own workbook page and was accompanied by 10 Biblical questions associated with the location as well as Old/New Testament verses for finding answers. Each location page also included historical facts and trivia; information about the location’s current population, government, economy, and points of interest; and several images of the archeological record and landscape of that place. Although many participants were Hispanic, we did not specifically culturally tailor workbook content. Three clergy checked the workbook for face and content validity, and based on their feedback we modified the workbook accordingly. School principals also reviewed the workbook for appropriateness prior to consenting their schools to participate.

We awarded one toe token (fitnessfinders.net) to students for every 5000 steps taken or 50 questions correctly answered. We also awarded students who completed the virtual journey an Easter-themed bookmark.

### Procedure

At each school during January/February 2017, the lead author met students at an assembly to introduce the study, demonstrate proper pedometer wear, answer questions, and distribute parent consent and student assent forms. At a follow-up assembly held approximately 1 week before the start of Lent we reminded participants returning signed assent and consent forms how to wear the pedometer and of the purpose and ideal mindset to achieve step goals, and instructed them how to record and graph steps. We further explained that they should go about their normal movement routine during the 2-week control condition (i.e. no *Savior’s Sandals* workbook; wearing pedometer and recording step counts).

Thereafter participants’ primary classroom teachers took responsibility for daily management of study protocol, which included distributing and collecting pedometers each day and reminding their students to record and graph daily steps in personal logs. We encouraged teachers to incorporate the content of and recording in the workbook into their religious studies and mathematics lessons, respectively. Although this was not compulsory and we did not formally check on the teachers’ follow-through—as the intervention was primarily focused on participants’ mindfulness and agency—anecdotally several teachers informed us that they were incorporating the materials and appreciated them. Each week we recorded the number of questions each participant attempted (voluntary, bonus steps awarded), which served as a crude proxy for fidelity to the workbook component of the intervention (see Table 2). Based on this measure, Schools 1, 3, and 2 were least to most compliant, respectively.

**Table 2:** Characteristics of schools in relation to workbook completion over the 4-week treatment (*n* = 3)

School	% students completing ≥1 questions/Mean (SD) questions attempted			
	Week 1	Week 2	Week 3	Week 4
1	0.0%/0.0 (0.0)	40.0%/1.3 (0.4)	0.0%/0.0 (0.0)	2.9%/1.0 (0.0)
2	100.0%/12.7 (5.7)	86.0%/16.2 (9.5)	54.0%/7.9 (5.4)	86.0%/15.0 (9.0)
3	76.7%/10.3 (2.8)	81.4%/14.8 (9.8)	46.5%/15.0 (9.6)	39.5%/10.8 (3.4)

*Note*. Due to a clerical error data for School 4 was lost. Mean (SD) calculated only for participants who completed at least one question

### Physical activity measure

For measuring step counts we used the Yamax Digiwalker SW-200 pedometer, which is considered a research-grade pedometer [26] and has been widely used in and validated for studies of children’s PA [27]. We tested a 10% random sample of pedometers to ensure that they measured steps within 20 ± 1 steps in a short walking test [26]. Participants recorded their daily step count while at school only on a paper log. A research assistant visited an assigned school each Friday or Monday to record step counts accumulated over the previous week. We averaged daily step counts across the 2- and 4-week periods that comprised control and treatment conditions, respectively, to yield control and treatment step count values for analysis.

### Data analysis

Descriptive statistics and the missing data pattern were computed using maximum likelihood estimation in PROC MI in SAS 9.4 for Windows (Cary, NC). School 1 (*n* = 35 students), School 2 (*n* = 50), School 3 (*n* = 43), and School 4 (*n* = 59) had 3.1–21.9%, 2.0–68.0%, 14.0–69.8%, and 10.2–69.5% of observations, respectively, missing during a single day across the 30-day study. Across all schools, there were a total of 5280 observations, with 3494 used and 1786 (33.8%) not used in the mixed model due to missing data.

General linear mixed models using PROC MIXED in SAS 9.4 for Windows (Cary, NC) with restricted maximum likelihood estimation to compensate for the missing data were used to compute the treatment effects on mean daily steps [28]. A fixed-effect only model was tested, and the significant residual covariance estimate indicated potential for clustered data. Therefore a second model was created that included school and student as random effects. The random effects model had a lower Akaike’s Information Criteria (AIC = 66,194.7 vs. 67,450.3 for the fixed effect model), indicating the inclusion of random effects improved model fit. Additionally, significant covariance estimates of student nested within school (*z* = 8.00, *P* < 0.001) and for the repeated measures of students (*z* = 40.83, *P* < 0.0001) suggested that estimates of fixed effects should be adjusted for random effects due to potential clustering of steps within schools [28].

## Results

Type 3 tests of the main effect after adjustments of estimates due to random effects, indicated a significant difference across schools (*F*_3, 150_) = 12.60, *P* < 0.0001) (Table 3), and grades (*F*_2, 3333_) = 6.93, *P* < 0.0001) (Table 4). Additionally, a significant main effect between treatment conditions (*F*_1, 3333_) = 23.42, *P* < 0.0001), and a significant interaction between school and treatment (*F*_3, 3333_) = 26.06, *P* < 0.0001) was found. No significant main effect for gender was found (boys, Mean = 7115, SE_m_ = 315; girls, Mean = 6306, SE_m_ = 259; Mean_diff_ = 809, SE_diff_ = 417; *t*_3331_ = 1.94, *P*_*adj*_ *=* 0.052), and no significant interaction between gender and treatment was found.

**Table 3:** Least-squared means of typical steps/day between control and treatment phases across schools

	School 1		School 2		School 3		School 4	
	Mean	SE	Mean	SE	Mean	SE	Mean	SE
Control	4585	466	7400	424	5789	437	8005	383
Treatment	4661	439	7183	395	7945^a^	424	8150	381

*Note.* Least-squared means are predicted population marginal means adjusted for unbalanced cell sizes with standard errors adjusted for model covariance parameters. Superscript ^a^ indicates a significant difference in the mean number of steps compared to the control condition (*P* < 0.01)

**Table 4:** Least-squared means of typical steps/day between control and treatment phases across grades

	Grade 6		Grade 7		Grade 8	
	Mean	SE	Mean	SE	Mean	SE
Average	7650^a^	368	6467	309	6014	371
Control	6811	388	6517	327	5921	396
Treatment	8489^b^	374	6418	315	6107	376

*Note*. Least-squared means are predicted population marginal means adjusted for unbalanced cell sizes with standard errors adjusted for model covariance parameters. Superscript ^a^ indicates a significant mean difference between grade 6 and grades 7 and 8 participants (*P* < 0.05). Superscript ^b^ indicates a significant difference in the mean number of steps compared to the control condition (*P* < 0.01)

Follow up tests of least-squared means with Bonferroni adjustments for multiple comparisons identified that only School 3 participants experienced a treatment effect accumulating significantly more steps in the treatment phase (Table 2). Additionally, follow up tests of least-squared means with Bonferroni adjustments for multiple comparisons identified that on average, grade 6 participants accumulated significantly more steps than grade 7 participants (Mean_diff_ = 1138, SE_diff_ = 476; t_3331_ = 2.48, *P*_*adj*_ *=* 0.020) and grade 8 participants (Mean_diff_ = 1636, SE_diff_ = 528; *t*_3331_ = 3.10, *P*_*adj*_ *=* 0.003). There were no significant differences in the typical daily step count for grade 7 versus grade 8 participants. Moreover, only grade 6 participants experienced a significant treatment effect (Mean_diff_ = 1678, SE_diff_ = 198; *t*_3331_ = 8.47, *P*_*adj*_ *<* 0.001). (See Table 4 for grade level comparisons.)

## Discussion

Our study’s primary objective was to determine if a faith-based pedometer intervention, predicated on students walking in the footsteps of Jesus by virtually following his life path, could increase steps during Lent. We observed a main treatment effect for School 3 only (*d* = 0.9). This school was allocated to the control condition for the first 2 weeks and was comprised primarily of girls (83%). Surprisingly, School 3 had the smallest outdoor physical space (< 800 m^2^) for accommodating movement. It was, however, the only school with an official wellness policy—a 17-page document patterned after the CSPAP and available on its website that included sections formalizing PA policy and other activities that promote student wellness. The latter section’s verbiage included statements about (1) developing relationships with community partners—including universities—that support wellness policy implementation, and (2) integrating wellness activities across the entire school setting. Therefore while the propositions of faith, mindfulness, and SCT may have contributed to a treatment effect, strong PA policy and its implementation may also have been crucial to the success of the intervention at School 3.

Our study’s secondary objectives were to determine if treatment effects differed based on gender, grade level, and order of treatment. We observed similar treatment effects for boys and girls with boys starting slightly, though not statistically significantly, higher. In a study that infused religious themes and physically active learning experiences into Christian Sunday school curriculum, Trost et al. [29] did not include analyses of gender differences in PA (steps/min). In contrast, Kahan and Nicaise [14] (study upon which the present study was based) found boys accumulated more steps than girls under control conditions (PE days = + 2076 steps/day, *d* = 1.6; no-PE days = + 1011 steps/day, *d* = 1.8), and the magnitude of differences widened during treatment (PE days = + 3242 steps/day, *d* = 4.0; no-PE days = + 1520 steps/day, *d* = 2.0). Kahan and Nicaise [14] attributed gender differences in steps/day to culturally constrained opportunities for additional PA and a less physically active PE curriculum for Muslim female students. These were not issues in our study of Catholic students and we are heartened, that relative to step count, boys and girls responded similarly to the intervention.

We observed a main effect for grade level with 6th grade, compared to 7th and 8th grade participants, significantly increasing steps/day compared to control conditions (+ 1678 vs. -99 (7th) and + 186 (8th)). In a pooled analysis of 26 longitudinal studies of PA level among adolescents between ages 10–19 years, Dumith et al. [30] found that PA level declined 7% annually. Thus, it is possible that 6th graders, generally, would be expected to accumulate more PA than students in higher grades. That these 6th graders also demonstrated a treatment effect may partially relate to them transitioning stages of faith between elementary and middle school grades for which they interpreted the directive to walk in the footsteps of Jesus more literally than older students who filter religious content through a more nuanced, socioecologically-informed lens [19]. Relative to SCT, in a study of 363 5th–8th graders, social support by peers but not parents was positively associated with PA levels [31]. Beyond the source of support, younger children perceived increased support from peers and parents for doing activity with them than older children [31]. Thus, 6th graders in our study may have relied more on social facilitation of PA behavior than older children, which we encouraged from the outset.

There was no clear evidence that order of treatment mattered. School 3—the only school that demonstrated a significant treatment effect—was in the treatment-delayed school pairing along with School 4 (+ 145 steps/day), while Schools 1 (+ 76 steps/day) and 2 (− 217 steps/day) received treatment immediately. Increases in steps/day for the delayed-treatment schools occurred after the novelty of wearing a pedometer had most likely worn off, which is encouraging. Wearing a pedometer and tracking steps before the start of Lent and then transitioning to the *Savior’s Sandals* workbook may be an optimal strategy.

### Limitations

Our study abided by several recommendations Bopp et al. [10] made for future study of faith-based PA interventions: focus on youth, rigorous design, large sample size, objective measures of PA, and the reporting of setting-level details. While significant differences in mean steps/day were observed, not all students or schools responded the same way to the intervention (Tables 3 and 4). We offer several potential explanations. First, response differences could be due to how different ages reacted to the treatment; more consistent results may have been seen with differential treatment approaches based upon grade level. Second, there was no washout period as is usually seen in a cross-over design, so it is possible that the lack of a significant mean difference in steps/day for Schools 1 and 2 (treatment first) was due to the residual effects of the treatment. We felt unable, however, to include a washout period given that the span of Lent is fixed at 6 weeks and the minimum recommended intervention duration for pedometer studies is 4 weeks [32]. Third, School 4 reported a higher mean for steps than the other schools during both the control and treatment, which could indicate that they: (1) had no room to increase their steps within the context of a regular school day (i.e. a ceiling) or (2) were already walking more due to other reasons. Indeed School 4 was in session until 5 pm daily, providing an extra ≥1.5 h of time for movement compared to other schools; and outdoor space available for movement at School 4 exceeded 9200 m^2^, which was far larger than at any other school.

Additionally, the percentage of observations missing from the analysis may potentially limit the strength of conclusions. We attempted to adjust and control for missing data by using restricted maximum likelihood estimation and the mixed-model procedure that is well-suited for handling missing data [33]. Moreover, reporting and recording pedometer data may be variable across students, teachers, and schools, which may also introduce additional unintended variance in the data. Nonetheless, we are confident in our conclusions based upon our analyses of the data at hand. (In general, missing data and reporting accuracy is always a potential limiting factor in large-scale field trials.)

PA measured by the Yamax Digiwalker reflects a combination of light PA and MVPA. We were not able to separate the two intensity levels. Pedometers that are able to do so (e.g. Walk4Life MVP) have additional buttons and settings that are more difficult for students of this age to reliably use. Nonetheless, light PA is preferable to sedentary activity and confers different health benefits than MVPA.

We were not formally aware of whether or how classroom teachers may have minimized or augmented *Savior’s Sandals* content during math or religious studies lessons. Additionally, we did not ask students when or how they additionally moved during the study’s treatment phase. Additional study of these issues may identify means that allow for potentiation of program effects. We presented data (Table 2) that suggested students at two schools—including School 3 (which demonstrated a treatment effect)—were regularly and moderately-to-highly engaged with the *Savior’s Sandals* vis-à-vis workbook questions answered. A more comprehensive process evaluation should be conducted in future studies. The materials we used for our intervention our scalable and capable of being widely disseminated. Pedometers are relatively inexpensive and workbook and recording materials can be set up on line. Beyond students, many others in the faith community could participate in programs such as ours without additional modifications to the materials we provided. Specifically, school staff, parents, clergy, and the Catholic community at large all observe Lent to varying degrees. As well, a diocesan bishop has the capacity to issue proclamations, and as such could promote programs like *Savior’s Sandals*/Physical Activity for Lent.

## Conclusions

Children can spend 50% or more of their waking hours at school, therefore it is incumbent on schools to provide students with sufficient opportunities to accumulate recommended levels of PA. The CSPAP advocates a mixture of PE, recess, classroom activity, before- and after-school PA programs, active transportation to/from school, and intra- and extramural sport [6]. Promotion of PA through faith-based, self-regulated mindful movement—in lieu of sedentary behavior—throughout the school day provides an additional option that is unrelated to the CSPAP components.

The merging of faith into religious school settings supports two domains identified in the National Physical Activity Plan [9]. To wit, a Christian curriculum intervention resulted in the intervention group accumulating a significantly greater step/min rate during Sunday school than in the control group (*d* = 2.9) [29]. Results of our study, which tracked PA across a much longer school day and its curriculum, and which was student-centered versus teacher-directed, suggest that younger students and PA-supporting schools responded most positively to the *Savior’s Sandals*. During lunch and recess discretionary periods, students in this age group may accumulate a combined 23–25% of total steps during school hours [34]. During lunch, specifically, students in this age group reported walking as the most prevalent PA type with it contributing an average of 4.3 min of PA within an intensity range of 2.9–4.6 MET [35]. Thus, the *Savior’s Sandals* can provide an important source of ancillary PA and associated energy expenditure, during a recurrent religious observance that encompasses 17% of the school year, while also reinforcing religious instruction in the Catholic tradition.

## Abbreviations

CSPAP: Comprehensive School Physical Activity Plan
MET: Metabolic equivalent of task
MVPA: Moderate-to-vigorous physical activity
NPAP: National Physical Activity Plan
PA: Physical activity
PE: Physical education
SCT: Social cognitive theory
US: United States
WOS: Whole of school
